# Crystal Structure of *Talaromyces cellulolyticus* (Formerly Known as *Acremonium cellulolyticus*) GH Family 11 Xylanase

**DOI:** 10.1007/s12010-014-1130-9

**Published:** 2014-08-20

**Authors:** Misumi Kataoka, Fusamichi Akita, Yuka Maeno, Benchaporn Inoue, Hiroyuki Inoue, Kazuhiko Ishikawa

**Affiliations:** Biomass Refinery Research Center, National Institute of Advanced Industrial Science and Technology (AIST), 3-11-32, Kagamiyama, Higashihiroshima, Hiroshima 739-0046 Japan

**Keywords:** X-ray crystal structure, Biomass, Xylanase, Hemicellulose, *Talaromyces cellulolyticus* (formerly known as *Acremonium cellulolyticus*), β-Jelly roll

## Abstract

*Talaromyces cellulolyticus* (formerly known as *Acremonium cellulolyticus*) is one of the mesophilic fungi that can produce high levels of cellulose-related enzymes and are expected to be used for the degradation of polysaccharide biomass. In silico analysis of the genome sequence of *T. cellulolyticus* detected seven open reading frames (ORFs) showing homology to xylanases from glycoside hydrolase (GH) family 11. The gene encoding the GH11 xylanase C (TcXylC) with the highest activity was used for overproduction and purification of the recombinant enzyme, permitting solving of the crystal structure to a resolution of 1.98 Å. In the asymmetric unit, two kinds of the crystal structures of the xylanase were identified. The main structure of the protein showed a β-jelly roll structure. We hypothesize that one of the two structures represents the open form and the other shows the close form. The changing of the flexible region between the two structures is presumed to induce and accelerate the enzyme reaction. The specificity of xylanase toward the branched xylan is discussed in the context of this structural data and by comparison with the other published structures of xylanases.

## Introduction

Cellulose and xylan are linked together in plant cell walls. Xylan is one of the major structural components of plant cell wall and the second-most abundant renewable resource biomass in nature. Xylan consists of xylose moieties polymerized via a series of β-1,4-xylosidic bonds. Xylanases (endo-1,4-β-xylanases; EC 3.2.1.8) catalyze the hydrolysis of β-1,4-xylosidic bonds of xylan, constituting a class of enzymes that are critical for the degradation of hemicellulosic polysaccharides in biomass. Based on amino acid sequence similarities, xylanases typically can be classified into either of two groups (family 10 and family 11) of glycoside hydrolases (GH; www.cazy.org/) [1–6]. The structures of GH10 and GH11 xylanases generally fold into (β/α)_8_-barrel and β-jelly roll structures, respectively. Filamentous fungi produce a wide spectrum of enzymes capable of degrading cellulose and xylan [7–9].

*Talaromyces cellulolyticus* (formerly known as *Acremonium cellulolyticus*; originally isolated by Yamanobe et al. in 1982) is a fungus displaying one of the highest known levels of cellulolytic enzyme production [10]. It was proposed that *A. cellulolyticus* is a new species, *Talaromyces cellulolyticus*, on the basis of morphology and molecular evidence [11]. Fujii et al. reported that the culture supernatant from *Talaromyces cellulolyticus* had a higher cellulose-specific activity and glucose yield from lignocellulosic materials than that from *Trichoderma reesei* [12]. Sequence analysis of the genome database of *Talaromyces cellulolyticus* revealed the presence of seven open reading frames (ORFs) with homology to the GH11 xylanase family [13]. Among these seven putative GH11 xylanases (Xyl11A, Xyl11B, Xyl11C, Xyl11D, Xyl11E, Xyl11F, and Xyl11G), Xyl11C (TcXylC) exhibited the highest specific activity toward xylan [13]. To our knowledge, a crystal structure has not previously been reported for a xylanase from *Talaromyces cellulolyticus*. In this paper, we report the cloning and overexpression of the gene encoding TcXylC, permitting us to solve the crystal structure and clarify its function.

## Methods

### Construction of the Truncated Enzyme

**Fig. 1:**
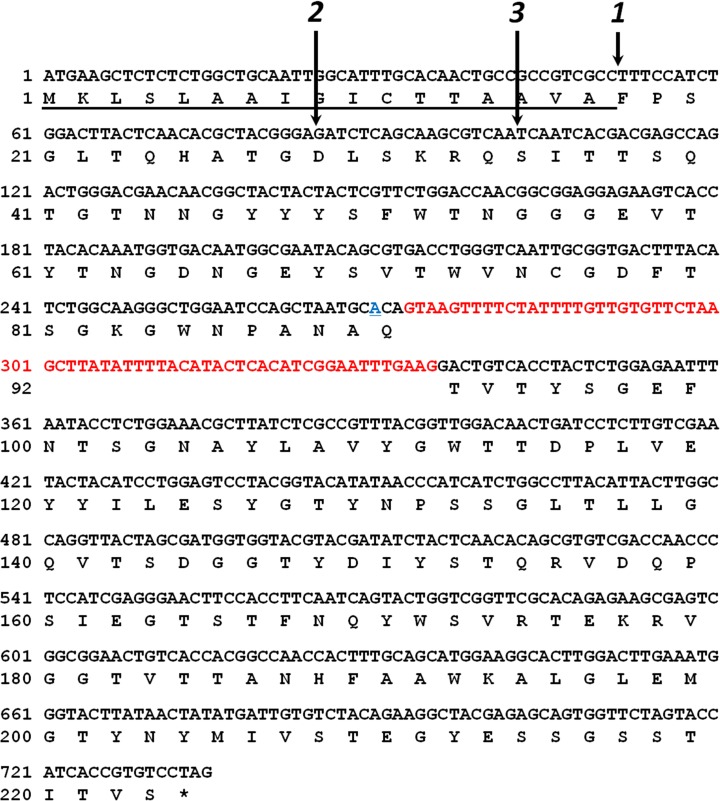
Deduced amino acid sequence for TcXylC protein. The putative signal peptide is *underlined*. The intron sequence is identified by the *red color*. The adenine residue (*blue*) was substituted for cytosine residue (silent mutation) because of the gene construction. The *arrows 1*, *2*, and *3* show the N-termini of TcXylCΔN17, cleaved TcXylCΔN17, and TcXylCΔN34, respectively (color figure online)

From the genome database of *Talaromyces cellulolyticus*, seven kinds of ORF showing the homology to GH11 xylanase (*xylA*, *xylB*, *xylC*, *xylD*, *xylE*, *xylF*, and *xylG*) were found [13]. According to analysis using *GENETYX* (www.genetyx.co.jp/), it was revealed that the gene (*xylC*) exhibits high homology of the xylanase from *Trichoderma reesei* and codes for 223 amino acid residues including intron and 17 putative signal peptides. The genome DNA of *Talaromyces cellulolyticus* strain CF-2612 [14] was purified by anion exchange resin column using Genomic-tip (QIAGEN). Two exon regions without signal sequence (sequence numbers 52-272, and 336-735 (Fig. 1)) of *xylC* gene were amplified using genome DNA. Using these fragments, the ORF (TcXylCΔN17) was constructed and inserted into the vector (pET-11a).

### Expression and Purification of TcXylCΔN17

A truncated enzyme (TcXylCΔN17) with 17 amino acid residues (putative signal sequence) that deleted and added Met residue at the N-terminal region of TcXylC was expressed and purified by the following method. The constructed expression plasmid vector described above was introduced into *Escherichia coli* strain BL21(DE3) for recombinant protein. Cells were grown in Luria-Bertani medium addition of 100 μg/mL ampicillin at 37 °C under continuous shaking. After the optical density at 600 nm (OD_600_) reached 0.6, protein expression was induced with 0.1 mM isopropyl-β-d-thiogalactopyranoside (IPTG) for 16-h incubation at 30 °C. Cells were harvested by centrifugation and pellets were frozen at −20 °C. After freezing, pellets were resuspended in Tris buffer (20 mM Tris-HCl (pH 8.0)), and suspension was centrifugation. Periplasmic fraction was purified by anion exchange chromatography using a HiTrap Q HP column (GE Healthcare) and a 0.0–1.0 M NaCl gradient in 20 mM Tris-HCl (pH 8.0). TcXylCΔN17 containing fraction was dialyzed with 20 mM Tris-HCl (pH 8.0) and concentrated using Amicon Ultra-15 (Millipore). The molecular weight and purity of the protein were checked by sodium dodecyl sulfate polyacrylamide gel electrophoresis (SDS-PAGE). The protein concentration of TcXylCΔN17 was determined from UV absorbance at 280 nm (A_280_), based on an extinction coefficient of 57,180 calculated from the protein sequence [15].

### Molecular Weight Determination and N-Terminal Amino Acid Sequencing

The molecular weights of purified enzymes were analyzed by matrix-assisted laser desorption–ionization/time-of-flight mass spectrometry (MALDI-TOF MS). The N-terminal amino acid sequences were analyzed by Edman degradation. Both analyses were carried out at APRO Science Inc. (Tokushima, Japan).

### Thermal Shift Assay

The protein thermal shift assay (TSA) [16] was performed using real-time PCR and monitored the protein denaturation using sensitive dye, SYPRO Orange dye. The purified protein was adjusted to the concentration of 0.5 mg/mL in 20 mM sodium acetate buffer (pH 5.5). Twenty microliters of purified protein was mixed with 1 μL of SYPRO Orange dye. The fluorescence data was plotted by the real-time PCR instrument throughout the temperature range from 25 to 85 °C; a melt curve was generated, and the midpoint or *T*_m_ of the resulting curve was taken as a reference of the thermal stability of a protein of interest.

### Crystallization

The purified TcXylCΔN17 was concentrated to 10 mg/mL and then dialyzed against 20 mM Tris-HCl (pH 8.0). The crystals of the TcXylCΔN17 were prepared and grown using a reservoir solution that consisted of 0.1 M Bis-Tris (2-[bis(2-hydroxyethyl)amino]-2-(hydroxymethyl)propane-1,3-diol) (pH 5.5), 0.8 M sodium dihydrogen phosphate, and 0.8 M potassium dihydrogen phosphate at 25 °C by the hanging-drop vapor-diffusion method with the drops composed of equal volumes (1.0 μL) of the protein and reservoir solutions equilibrated against 0.4 mL of reservoir solution.

### X-ray Data Collection

The selected crystals were placed in the cryoprotectant solution that consisted of 33 % (*v*/*v*) ethylene glycol, 0.67 M Bis-Tris (pH 5.5), 0.53 M sodium dihydrogen phosphate, and 0.53 M potassium dihydrogen phosphate. The soaked crystal was collected with a cryo-loop and flash-cooled under cryostream of nitrogen gas at −173 °C. X-ray diffraction data experiment was carried out at the SPring-8 BL44XU in Hyogo, Japan. The dataset was collected at a wavelength of 0.9 Å using a Rayonix MX225HE detector at the beamline. The distance from the crystal to the detector was 230 mm. The crystal was rotated at 200° with an oscillation angle of 0.5° per frame. The data collected from diffraction measurements were indexed, integrated, and scaled with the programs in the *HKL*-2000 software package [17].

### Structure Solution and Refinement

TcXylCΔN17 structure was solved by molecular replacement with *MOLREP* [18] in the *CCP4* package using as a search model the structure of XYNII from *Trichoderma reesei* (TrXyl) (Protein Data Bank (PDB; www.pdb.org/) ID 1XYP) [1] which shows 61 % sequence identity with TcXylCΔN17. The resulting electron density maps were used to refine and build a model of TcXylCΔN17. Structure model-building was performed with *Coot* [19]. The structure was refined using *REFMAC5* [20]. Water molecules were introduced at peaks over 3.0 root-mean-square deviation (RMSD) in the difference Fourier map fulfilling reasonable interactions with the protein model. Ramachandran plot of the final structure was validated using *ProCheck* [21]. Molecular graphics images were generated using *PyMOL* (www.pymol.org/). Processing parameters are presented in Table 1.

**Table 1:** Data collection and refinement statistics of the structure of TcXylCΔN17

Data set	
Data collection	
Wavelength (Å)	0.9
Space group	*P*2_1_
Unit-cell parameters	*a* = 40.51 Å, *b* = 60.11 Å, *c* = 106.98 Å, *β* = 99.07°
Molecules/asymmetry unit	2
Matthews coefficient (Å^3^/Da)	2.90
Solvent content (%)	57.3
Resolution range (Å)	50.0–1.98 (2.01–1.98)
Total number of observed reflections	125,936
Number of unique reflections	35,412 (1,763)
Average *I*/*σ*(*I*)	11.5 (3.3)
*R*_merge_^a^ (%)	10.8 (34.7)
Redundancy	3.6 (2.9)
Completeness (%)	99.4 (97.6)
Refinement	
Number of atoms	
Amino acid residues	2,922
Water	245
Resolution used in refinement (Å)	50.0–1.98
*R*_work_^b^/*R*_free_^c^ (%)	23.2/27.6
RMSD bond distance (Å)	0.022
RMSD bond angle (degree)	1.96
Mean overall *B*-factor (Å^2^)	25.79
Ramachandran plot	
In most favored regions (%)	95.5
In disallowed regions (%)	0.5
PDB ID	3WP3

^a^ *R*_merge_ = Σ_*hkl*_Σ_*i*_|*I*_*i*_(*hkl*) − <*I*(*hkl*) > | / Σ_*hkl*_Σ_*i*_ *I*_*i*_(*hkl*), where *I*_*i*_(*hkl*) is the intensity of the *i*th measurement of reflection *hkl*, including symmetry-related reflections, and < *I*(*hkl*) > is their average

^b^ *R*_work_ = Σ_*h*_Σ_*i*_||*F*_o_| − |*F*_*c*_|| / Σ|*F*_o_|

^c^ *R*_free_ is *R*_work_ for approximately 5 % of the reflections that were excluded from the refinement

## Results and Discussion

### Preparation of the Recombinant Enzyme

**Fig. 2:**
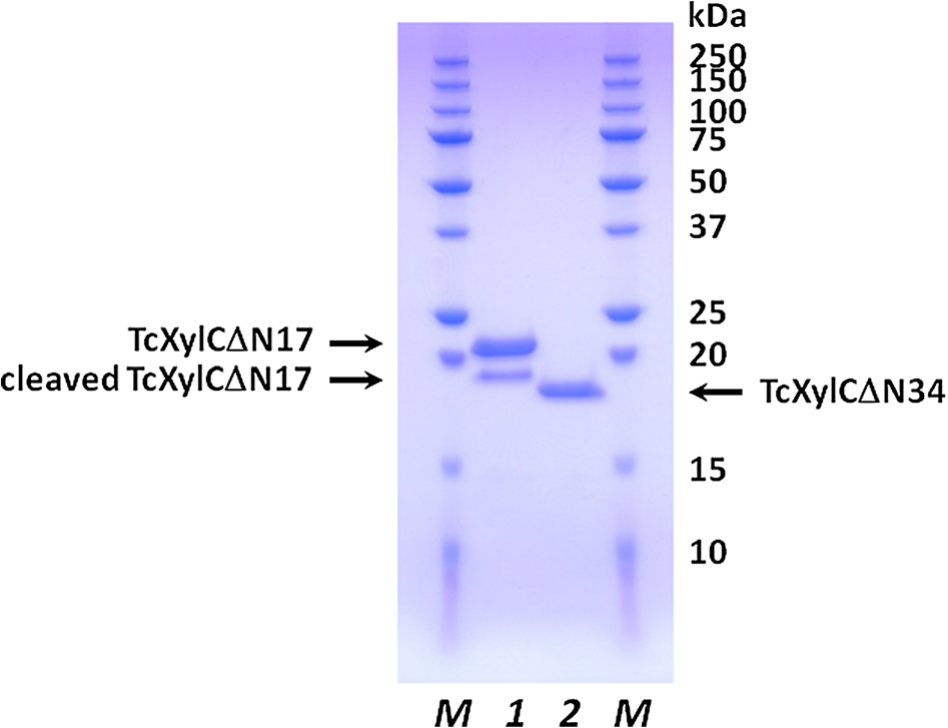
SDS-PAGE analysis of two purified TcXylC enzymes. *Lanes* designated *1* and *2* correspond to purified TcXylCΔN17 (not fresh) and TcXylCΔN34, respectively. Note that the TcXylCΔN17 preparation includes a shortened (cleaved) product. Molecular weight standards (*lane M*)

According to the analysis using *GENETYX* (www.genetyx.co.jp/), the putative TcXylC gene contains the intron and signal peptides (Fig. 1). An ORF encoding the putative mature enzyme (designated TcXylCΔN17), lacking the intron and signal regions, was constructed using PCR and inserted into the pET-11a expression vector. The recombinant enzyme was overproduced in *E. coli* using the pET system. The recombinant enzyme was prepared and purified as described in “Methods” section. The molecular weight of the purified TcXylCΔN17 was determined to be about 20 kDa by SDS-PAGE (Fig. 2). It was observed that the purified TcXylCΔN17 was cleaved to yield a smaller molecular weight species (smaller than 20 kDa; designated as cleaved TcXylCΔN17) over time. The 34 amino acid residues of N-terminus of the protein do not seem to be folded from the structural analysis of the xylanase (described below). Thereby, we constructed the enzyme that deleted 34 amino acid residues of N-terminus (TcXylCΔN34) (Fig. 1). The overproduction and purification of TcXylCΔN34 provided yield similar to those seen for TcXylCΔN17. SDS-PAGE analysis of TcXylCΔN34 revealed a stable single band, as shown in Fig. 2. Based on the amino acid sequences, the calculated molecular masses of TcXylCΔN17 and TcXylCΔN34 were predicted to be 22,485.21 and 20,660.20, respectively. In SDS-PAGE analysis, the cleaved TcXylCΔN17 ran slightly larger than TcXylCΔN34 (Fig. 2). The detailed molecular masses of the two species were determined by mass spectrometry. The results of MALDI-TOF MS analysis revealed that the total masses were 22,501.66 (TcXylCΔN17), 21,231.38 (cleaved TcXylCΔN17), and 20,525.83 (TcXylCΔN34). To determine the cleavage sites, we examined the N-terminal sequences of each of the proteins. These analyses showed that the N-terminal five amino acid residues of the enzymes were Met-Phe-Pro-Ser-Gly (TcXylCΔN17), Asp-Leu-Ser-Lys-Arg (cleaved TcXylCΔN17), and Ser-Ile-Thr-Thr-Ser (TcXylCΔN34), demonstrating that TcXylCΔN17 was cleaved between Gly28 and Asp29 (Fig. 1) following overproduction in the *E. coli* expression system. Enzyme assays revealed that the three recombinant enzyme preparations exhibited similar specific activities for xylanase.

### Thermostability of TcXylC

**Fig. 3:**
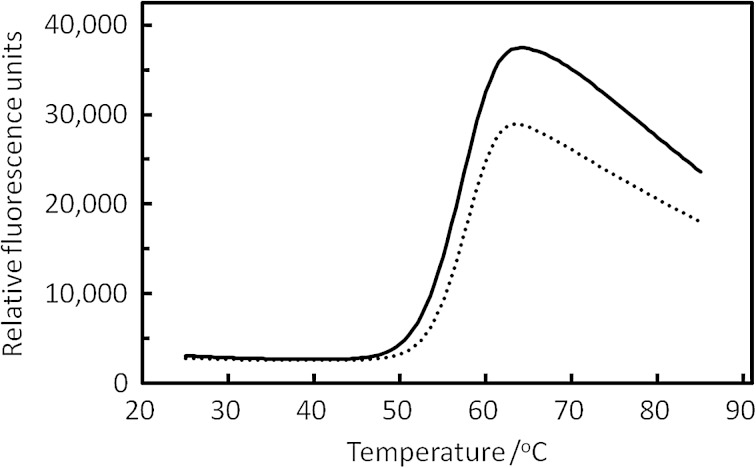
Thermal stability assay of TcXylC proteins. The thermal shifts are indicated for fresh purified TcXylCΔN17 (*solid line*) and purified TcXylCΔN34 (*dotted line*)

Thermostability of TcXylC was analyzed by TSA [16]. The result of TSA of the recombinant enzymes using the real-time PCR instrument is shown in Fig. 3. The *T*_m_ values of the TSA melting curve of purified fresh TcXylCΔN17 (not cleaved) and TcXylCΔN34 were 57.5 and 58.0 °C, respectively. The similarity of the *T*_m_ values indicates that the cleavage of N-terminus (17 amino acid residues from N-terminus of TcXylCΔN17) was unrelated to protein folding or the thermostability of the enzymes.

### Structural Determination

**Fig. 4:**
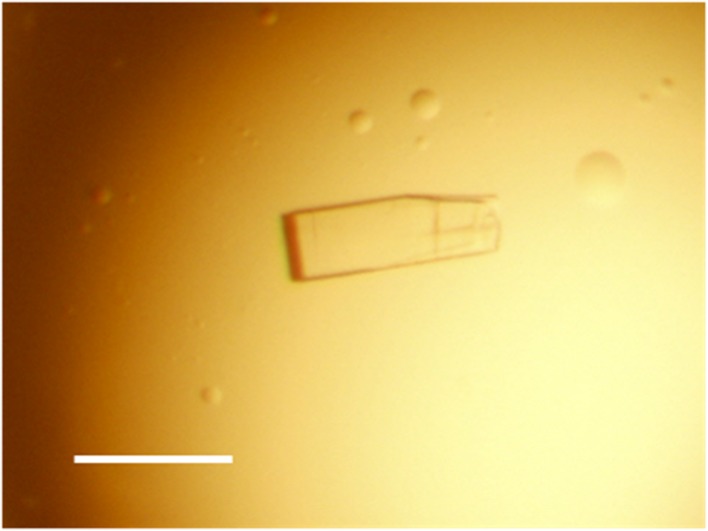
A photograph of the TcXylCΔN17 crystals. The *scale bar* corresponds to 0.2 mm

Crystal of the recombinant xylanase (TcXylCΔN17) was prepared by the hanging-drop vapor-diffusion method. After optimization of the crystallization conditions, crystals of average size 0.1 mm × 0.2 mm × 0.02 mm (Fig. 4) were obtained within 3 days at 25 °C using a reservoir solution consisting of 0.1 M Bis-Tris (pH 5.5), 0.8 M sodium dihydrogen phosphate, and 0.8 M potassium dihydrogen phosphate. The statistics of data collection and refinement are summarized in Table 1. Diffraction data were collected to a resolution of 1.98 Å. The crystal belonged to a space group *P*2_1_ with unit-cell parameters *a* = 40.51 Å, *b* = 60.11 Å, *c* = 106.98 Å, *β* = 99.07°. The TcXylCΔN17 structure was solved by molecular replacement with *MOLREP* [18] in the *CCP4* package using as a search model the structure of TrXyl (PDB ID 1XYP) [1]. In the asymmetric unit, two molecules of TcXylCΔN17 were observed. The presence of two enzyme molecules in the asymmetric unit gives a crystal volume per protein mass (*V*_M_) of 2.90 Å^3^/Da and a solvent content of 57.3 % (*v*/*v*) [22]. After the refinement, the *R* factors were estimated to be *R*_work_ = 23.2 % and *R*_free_ = 27.6 %.

### Overall Structure of TcXylC

**Fig. 5:**
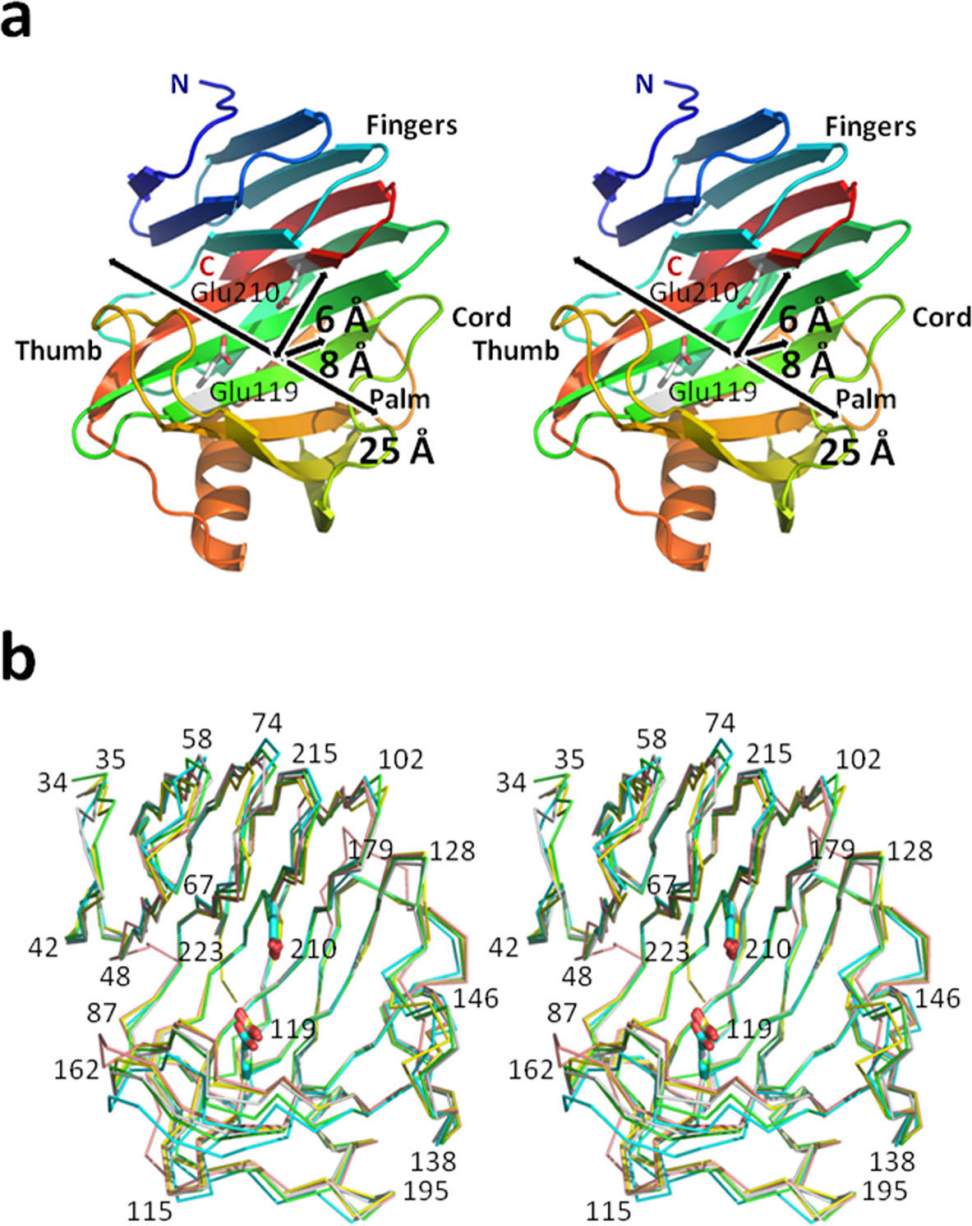
Overall crystal structure of TcXylCΔN17 drawn with wall-eyed stereo view. **a** Ribbon model viewed from front is color-graduated according to residue number from N-terminus with *blue* to C-terminus with *red*. The *arrows* show size of the active site cleft. Length, width, and depth are 25, 6, and 8 Å, respectively. Two of the active site residues (Glu119 and Glu210) are shown with bond model. **b** The model of TcXylCΔN17 structure is superimposed with other GH family 11 xylanase structures. The distinct xylanase structures are identified by *color*: *green* (TcXylCΔN17), *cyan* (TrXyl), *gray* (CtXyl), *yellow* (NpXyl), and *magenta* (BcXyl). Selected residues of TcXylCΔN17 are indicated by *number* (color figure online)

**Fig. 6:**
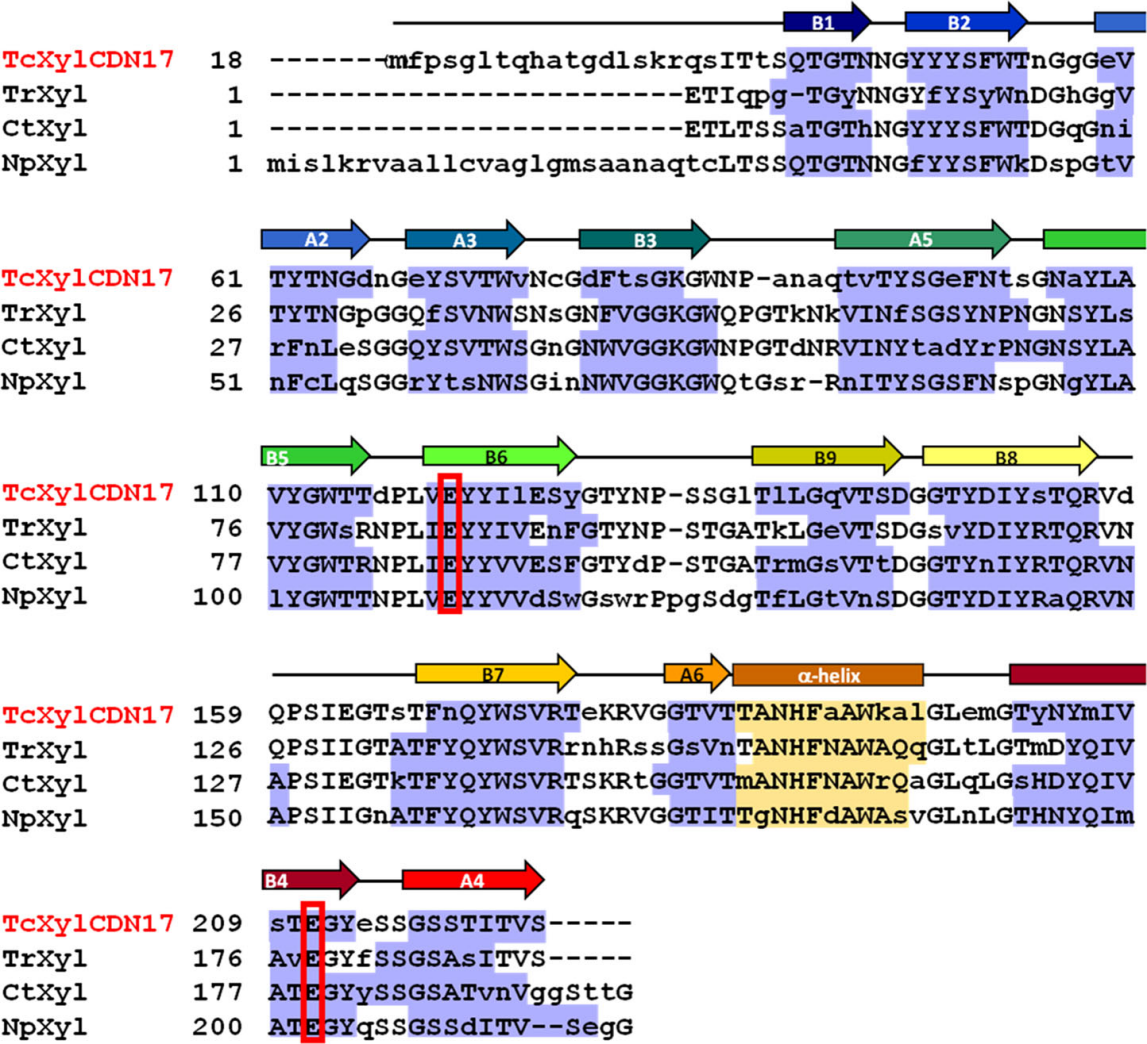
Structure-based sequence alignment of TcXylCΔN17 with TrXyl, CtXyl, and NpXyl. Residues that show structural similarity are *capitalized*. The catalytic residues are indicated with *red box*. The β-sheets are colored *blue boxes*. The β-sheets in the TcXylCΔN17 structure are labeled *A* and *B*. Respective strands are labeled (*A2*–*A6* or *B1*-*B9*) according to their positions in the two β-sheets. The α-helix is *colored yellow boxes* (color figure online)

The model of TcXylCΔN17 was able to be built from Gln34 to Ser223. The structure of the peptide sequence from N-terminus to Arg33 was not observed. The disordered region (from Met1 to Arg33) seems to be the signal sequence of the xylanase [13]. The overall structure of TcXylCΔN17 shows a β-jelly roll fold, as seen in other GH11 family xylanases (Fig. 5) [1–6]. TcXylCΔN17 consists of 14 β-sheets (labeled A2 to A6 and B1 to B9) and one α-helix (Figs. 5a and 6). The β-sheets were designated as A2 (residues 58–65), A3 (residues 68–74), A4 (residues 216–223), A5 (residues 92–101), A6 (residues 181–184), B1 (residues 40–44), B2 (residues 47–53), B3 (residues 78–83), B4 (residues 201–212), B5 (residues 104–114), B6 (residues 118–126), B7 (residues 167–174), B8 (residues 146–155), and B9 (residues 136–144). The α-helix corresponds to residues 185–195. The shape of the overall GH11 family xylanase structure has been described as a “right hand” [1, 5]. The long loop between B7 and B8 forms a “thumb.” The β-sheets A and B form “fingers.” The β-sheet B and the α-helix form a “palm.” By analogy to other GH11 family xylanases, it was clarified that two active site residues, Glu119 and Glu210, are located in this region. The loop between B6 and B9 forms a “cord.” The structurally determined N-terminus of TcXylCΔN17 (from Gln34) corresponds to the “little finger” of fingers region, and C-terminus is located at A4, between A3 and A5. The main chain structure of TcXylCΔN17 shows high similarity with the other GH11 xylanases for which structures have been solved, including TrXyl [1], xylanase from *Chaetomium thermophilum* (CtXyl) [2], xylanase 11A from *Neocallimastix patriciarum* (NpXyl) [3], and xylanase from *Bacillus circulans* (BcXyl) [4] (Fig. 5b). According to sequence alignment, TcXylCΔN17 exhibits high homologies of primary and secondary structures with these previously described xylanases (Fig. 6). Comparisons among the tertiary structures are expected to provide clues to the function of enzymes, such as the manner of substrate binding.

### Active Site Cleft

**Fig. 7:**
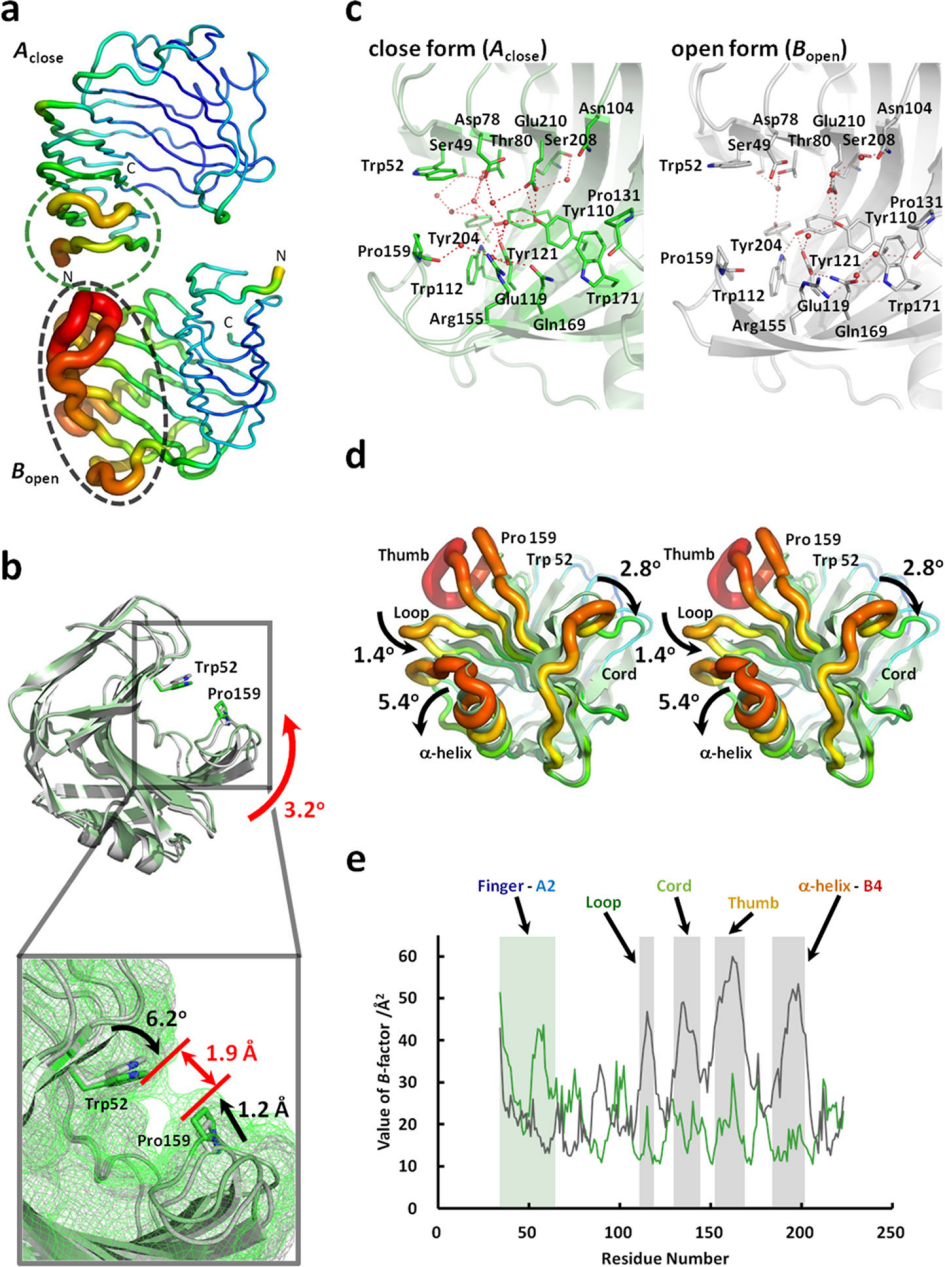
Structural comparison of *A*_close_ and *B*_open_ crystal forms. **a** The *B*-factor of amino acid residues of TcXylCΔN17. *Red* and *blue ribbons* indicate high and low *B*-factor amino acid residues, respectively. **b** Image showing superposition of *A*_close_ and *B*_open_ crystal forms. Surfaces of both subunits are drawn by mesh. **c** Hydrogen bond networks in the active site cleft. *A*_close_ and *B*_open_ are identified by *color*: *green* (*A*_close_) and *gray* (*B*_open_). **d** Wall-eyed stereo views of the structural comparison of *A*_close_ and *B*_open_. The structures are viewed from the *bottom side. A*_close_ and *B*_open_ are drawn by cartoon and ribbon models, respectively. In *B*_open_, *red* and *blue ribbons* show high and low *B*-factor amino acid residues, respectively. **e** *A*_close_ and *B*_open_ are identified by *color*: *green* (*A*_close_) and *black* (*B*_open_) (color figure online)

The active site cleft is approximately 25 Å long, 6 Å wide, and 8 Å deep in close form (Fig. 5a). The two catalytic residues, nucleophile Glu119 in β-sheet B6 and proton donor Glu210 in β-sheet B4, are located in this cleft. As noted above, two individual molecules exist in the asymmetric unit and are designated here as *A*_close_ and *B*_open_ in Fig. 7a. The RMSDs of the Cα atoms between *A*_close_ and *B*_open_ were estimated to be 0.55 Å, and differences in conformational regions were observed between *A*_close_ and *B*_open_. The thumb in *A*_close_ represents the close form (closed active site cleft), while the thumb in *B*_open_ represents the open form (open active site cleft). The RMSD value of the Cα atoms for the thumbs between *A*_close_ and *B*_open_ is 0.78 Å. According to superposition of fingers, the thumb of *A*_close_ inclines at 3.2° and close to fingers. Furthermore, the side chain of Trp52 inclines at 6.2°, and the main chain of Pro159 draws apart by 1.2 Å (Fig. 7b). In the active site of *A*_close_, the distance between Trp52 and Pro159, forming a tunnel, is 1.9 Å. In the cleft of *A*_close_, 10 water molecules were observed, and a network of 31 hydrogen bonds were formed between the water molecules and 12 amino acid residues (Ser49, Asp78, Thr80, Tyr110, Trp112, Glu119, Tyr121, Arg155, Pro159, Tyr204, Ser208, and Glu210) (Fig. 7c). In contrast, the cleft of *B*_open_ included 7 water molecules and a network of 21 hydrogen bonds formed between the water molecules and 14 amino acid residues (Ser49, Thr80, Asn104, Tyr110, Trp112, Glu119, Tyr121, Pro131, Arg155, Gln169, Trp171, Tyr204, Ser208, and Glu210) (Fig. 7c). Because the cross-sectional hydrogen bond networks are formed in the active site cleft, thumb gets close to finger that is formation of the tunnel. In the cleft of *A*_close_, 10 water molecules observed seem to mimic the substrate analog. On the other hand, in *B*_open_, the region from α-helix to B4 (residues 185–204) inclined at 5.4°, and the loop between B5 and B6 (residues 114–118) and cord wrapping thumb (residues 131–143) were spread at 1.4° and 2.8°, respectively, to outside of the enzyme by comparison with *A*_close_ (Fig. 7d). The average values of the overall *B*-factors in *A*_close_ and *B*_open_ were 20.9 and 29.3 Å^2^, respectively. The value of the *B*-factor in the thumb in *B*_open_ was 54.0 Å^2^. In *B*_open_, the values of the *B*-factor of the region from the finger to A2 (residues 34–65), the loop between B5 and B6 (residues 114–118), cord (residues 131–143), and the region from the α-helix to B4 (residues 185–204) are 31.8 Å^2^ (RMSD 0.73 Å), 42.3 Å^2^ (RMSD 0.24 Å), 42.3 Å^2^ (RMSD 0.68 Å), and 41.5 Å^2^ (RMSD 0.35 Å), respectively (Fig. 7e). Because the values of the *B*-factors of the finger region in *A*_close_ and thumb region in *B*_open_ exhibit high values (Fig. 7d, e), these regions are able to migrate, providing flexibility to the individual structures. These results suggest the following properties. For forming the enzyme–substrate complex, the flexible thumb effectively binds to or incorporates the substrate in *B*_open_. On the other hand, the flexing of the finger region in *A*_close_ facilitates the release of product from the enzyme–substrate complex. The changing of the flexible region between forms *A*_close_ and *B*_open_ is presumed to induce and accelerate the enzyme reaction.

### Substrate Binding

**Fig. 8:**
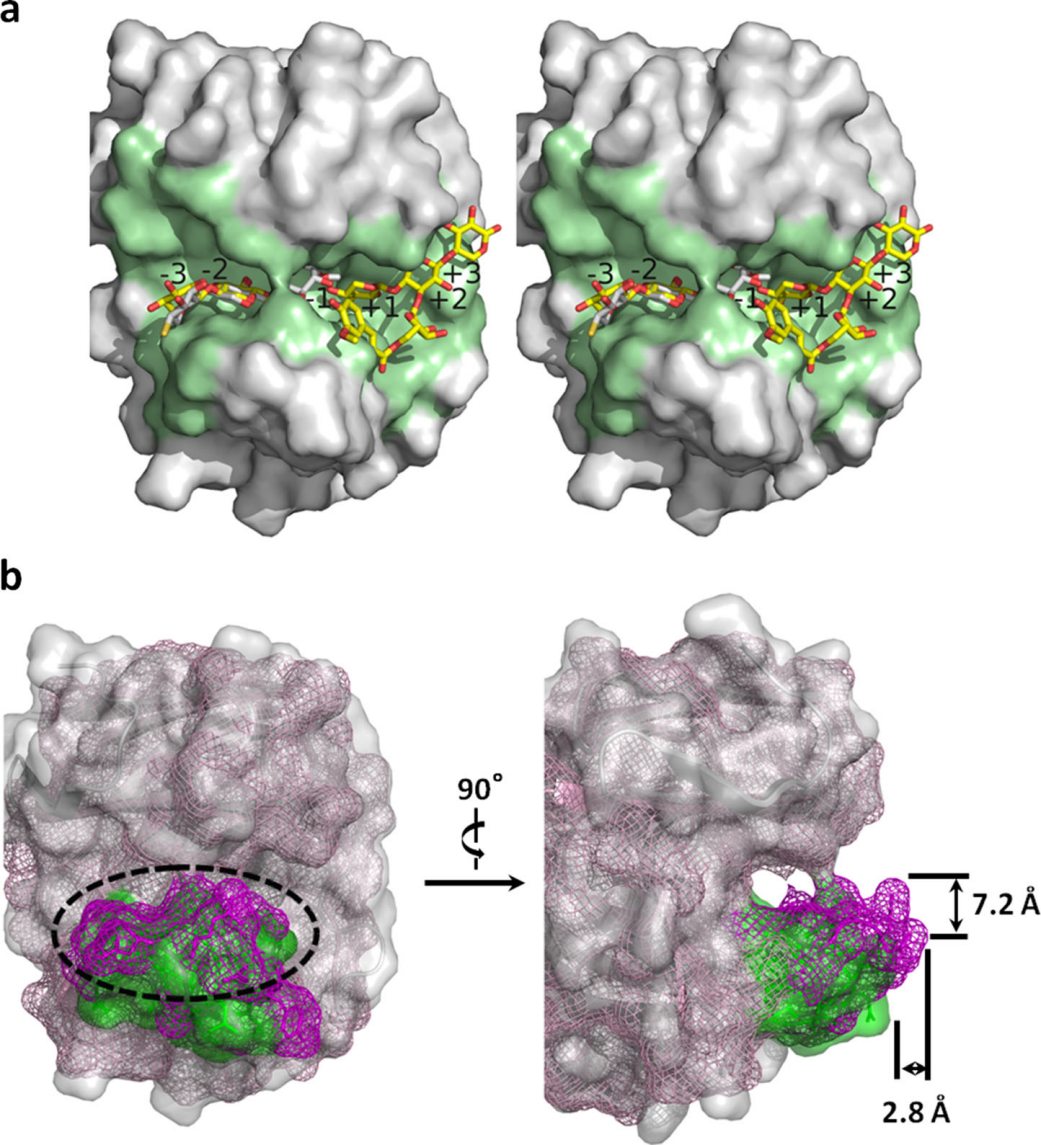
Structure comparison of TcXylCΔN17 and other xylanases. **a** Surface of the substrate-binding cleft of TcXylCΔN17 drawn with wall-eyed stereo view in *green*. Stick models of inhibitors are colored *gray* (CtXyl) or *yellow* (NpXyl). **b** Superposition of TcXylCΔN17 and BcXyl viewed from the front and the side to represent the active site. The surfaces of TcXylCΔN17 and BcXyl are identified by *color*: *gray* (TcXylCΔN17) and *pale magenta* (BcXyl). The “thumb” regions of TcXylCΔN17 and BcXyl are identified by *color*: *green* (TcXylCΔN17) and *magenta* (BcXyl) (color figure online)

The active site conformation of TcXylCΔN17 is highly homologous to that of TrXyl [1] (sequence identity 61 %, PDB ID 1XYP, Z-score 33.7), CtXyl [2] (sequence identity 59 %, PDB ID 1XNK, Z-score 34.8), NpXyl [3] (sequence identity 58 %, PDB ID 2VGD, Z-score 34.3), and BcXyl [4] (sequence identity 51 %, PDB ID 1XNB, Z-score 29.1). In TrXyl, the active site cleft of TrXyl has five xylose residue binding sites (subsites), from subsite −2 to +3. According to superposition of TcXylCΔN17 and TrXyl, it is estimated that the five xylose binding sites in TcXylCΔN17 are constituted as follows: subsite −3 (Ile161, Glu162, Gly163), subsite −2 (Ser49, Trp52, Tyr110, Trp112, Pro159, Ser160, Tyr204), subsite −1 (Asp78, Phe79, Thr80, Tyr110, Glu119, Tyr121, Arg155, Glu210), subsite +1 (Asp78, Tyr106, Tyr121, Arg155, Gln169, Trp171, Glu210), subsite +2 (Asn104, Tyr129, Tyr212), and subsite +3 (Tyr129). Superposition of TcXylCΔN17 and substrate analogs complexed in CtXyl and NpXyl is shown in Fig. 8a. The substrate analog for CtXyl covers the subsites from −3 to −1 in TcXylCΔN17. The substrate analog for NpXyl covers the subsites from −3 to −2 and from +1 to +3 in TcXylCΔN17. Focusing on the active site cleft of TcXylCΔN17, it is estimated that the large space opens from subsite −3 toward the nonreducing end and from subsite +2 toward the reducing end. According to the superposition, TcXylCΔN17 has enough space to accommodate some branched sugar chain substrates. On the other hand, BcXyl has a large “hump” in front of the active site (Fig. 8b). The hump in BcXyl is approximately 7.2 Å wide and 2.8 Å deep. In BcXyl, the main chain of the thumb, including bulky residues (Asp121 and Arg122), is 2.6 Å apart from that in TcXylCΔN17. According to superposition of TcXylCΔN17 with BcXyl, TcXylCΔN17 is expected to be able to bind some branched sugar chain substrates more easily than BcXyl does.

## Conclusion

From the genome database of *Talaromyces cellulolyticus*, seven kinds of ORF showing the homology to xylanase (GH11) were found. One of the recombinant enzymes, TcXylC, was crystallized, and the crystal structure was solved up to the resolution of 1.98 Å. In the asymmetric unit, two kinds of xylanase structures were observed: One shows an open form and the other shows a close form. This conformational change is induced in hydrogen bond networks between thumb and finger (Fig. 7c). Furthermore, because the values of *B*-factors of finger region in *A*_close_ and thumb region in *B*_open_ exhibit high values (Fig. 7d, e), these regions are able to fluctuate and are flexible. These results suggest that the tunnel has an open form in apo form as *B*_open_, and if the substrate binds to the active site cleft, the tunnel (close form) forms by replacing water molecules in *A*_close_. The flexibility of the active site cleft plays a role in substrate binding and enzyme activity. From the structural comparison of xylanase, it was clarified that changing the flexible region between two different forms induces and accelerates the enzyme reaction.
